# The Human Exersome Initiative (HEI): Rationale, study design, and protocol

**DOI:** 10.1371/journal.pone.0326149

**Published:** 2025-06-25

**Authors:** Daria Neyroud, Julia Primavesi, Guia Tagliapietra, Chantal Daucourt, Hector Gallart-Ayala, Raphael Gottardo, Julijana Ivanisevic, Aaron L. Baggish

**Affiliations:** 1 Institute of Sport Sciences (ISSUL), University of Lausanne, Lausanne, Switzerland; 2 Department of Cardiology, Lausanne University Hospital, Lausanne, Switzerland; 3 Metabolomics and Lipidomics Unit, Faculty of Biology and Medicine, University of Lausanne, Lausanne, Switzerland; 4 Swiss Institute of Bioinformatics, Lausanne University Hospital, Lausanne, Switzerland; University of Ljubljana, SLOVENIA

## Abstract

Habitual physical activity and exercise training (PA/EX) confers numerous health-benefits. Phenotypic adaptations and clinical health outcomes attributable to PA/EX are well-established, and molecular markers and mediators of the PA/EX response have been described. To date, the majority of prior work focused on the biochemical response to PA/EX has leveraged convenience samples of trained athletes participating in events, patients undergoing clinically indicated exercise stress testing, or laboratory protocols comprised of a single dose of exercise. Accordingly, the impact of “exercise dose”, defined by the product of intensity, duration, modality, and frequency, on the human biochemical response to PA/EX remains largely unexplored. The Human Exersome Initiative (HEI) was designed to fill this scientific knowledge gap. Specifically, the HEI will couple carefully controlled laboratory-based acute exercise testing with comprehensive systemic biochemical profiling to isolate the impact of PA/EX duration and intensity on human biochemistry. Herein, we describe the initial phase of the HEI which will aim to comprehensively define the impact of “exercise dose” on blood-based biochemistry in healthy young men and women. The overarching goal of the HEI is to elucidate how the human exercise response varies as a function of phenotypic variability. Using comparator data derived from young men and women, future iterations of this protocol will seek to determine how key sources of human variability (i.e., age, ethnicity, and the presence of comorbid disease) impact the biochemical response to PA/EX. We anticipate results from this work will facilitate biomarker discovery, the elucidation of molecular pathways and mechanisms associated with metabolic responses to exercise, and the identification of optimal exercise doses for future clinical interventions (i.e., tailoring preventive and therapeutic strategies).

## Introduction

The health benefits attributed to habitual physical activity and/or structured exercise (PA/EX) have been well documented. Numerous classic epidemiologic datasets have established a direct, curvilinear relationship between PA/EX and all-cause mortality, with the largest risk reduction occurring at the lowest end of the exercise dose spectrum [1]. Consequently, the steepest reductions along the mortality risk curve occur between sedentary people and those performing relatively low levels of PA/EX. Careful analysis of these classic epidemiologic datasets underlie contemporary physical activity guidelines. Current recommendations suggest at least 150 weekly minutes of moderate intensity (i.e., 3–6 metabolic equivalents, METs) exercise or 75 weekly minutes of vigorous intensity (i.e., > 6 METs) exercise as these values correspond to the nadir (i.e., the initial flattening) of the PA/EX-mortality curve [2–4].

The benefits of PA/EX extend beyond reductions in mortality risk. Several randomized controlled trials document positive disease-specific outcomes in response to PA/EX [5–8]. In addition, habitual PA/EX stimulates organ system-specific adaptations including enhanced cardiovascular reserve (i.e., increased maximal cardiac output and lower blood pressure) [9], favorable skeletal muscle morphology adaptations (i.e., higher mitochondrial content, mitochondrial function, and capillarization) [10–12], and lower levels of chronic systemic inflammation [13]. While these phenotypic responses to PA/EX have been firmly established, the molecular markers and mediators of these adaptions remain incompletely understood [14–16].

Prior work examining the acute molecular responses of PA/EX have largely leveraged convenience samples. Athletes participating in endurance sport competitions [17–21], and clinical populations undergoing clinically indicated exercise testing during routine medical care [20], have been used to identify biochemical pathways and individual molecules that respond to PA/EX. In addition, a small number of studies have examined organ-specific and/or systemic biochemical changes in response to a single dose of PA/EX [22–27]. At present, a large US-based, federally-funded, multi-center consortium is examining the molecular mechanisms of an exercise training intervention [15]. Specifically, the Molecular Transducer of Physical Activity Consortium (MoTrPAC) aims to provide comprehensive biochemical profile of the response to steady-state sub-maximal exercise [65% of peak oxygen consumption (V̇O_2peak_)] before and after a 12-week training intervention [16]. In aggregate, prior and ongoing work has and will continue to identify molecular families and individual molecules that are either up-regulated or down-regulated by PA/EX. However, the potential importance of “exercise dose” on biochemical regulation and corollary clinical outcomes remains largely unexplored.

“Exercise dose” is defined by the product of intensity, duration, and frequency. This framework mirrors the principal components of pharmaceutical dosing: drug potency, frequency of administration, and duration of therapy. The ability to effectively tailor pharmaceutical prescriptions at the individual patient level is enabled by the carefully scripted scientific sequence of drug development which relies on pre-clinical studies to elucidate mechanisms of action and phase I clinical trials to establish dose-response relationships and safety, while phase II and III clinical studies determine mechanisms of action and effectiveness across diverse populations. Analogous to classical Phase I clinical studies, comprehensive laboratory-based work characterizing dose-response relationships between multiple acute bouts of PA/EX and blood-based biochemical profiles are lacking. The Human Exersome Initiative (HEI) was designed to fill this scientific knowledge gap. Specifically, the HEI will couple carefully controlled laboratory-based exercise testing of variable intensities, durations, and modalities with comprehensive systemic biochemical profiling (i.e., proteomic, metabolomic, lipidomic, transcriptomic) to probe the impact of exercise dose on human biochemistry.

## Methods

### Study design

The HEI is a prospective, longitudinal, repeated-measures, human participants study examining the biochemical response to variable doses of acute exercise bouts. Participants will perform 10 experimental exercise sessions at pre-determined exercise doses as defined by individualized relative intensities and fixed absolute durations. Peripheral venous blood samples will be collected before (baseline), immediately after (post), and 60 min after (recovery) exercise completion for all sessions. Participant recruitment and data collection for the first phase of the HEI began on October 2^nd^, 2024 and is anticipated to reach completion by July 15^st^, 2025. Results from the biochemical analyses described below are anticipated to be available by the end of 2025. All participants will provide oral and written consent at the time of enrollment. All study procedures described below adhere to the Declaration of Helsinki and have been approved by the local institutional review board (CER-VD 2024-00525).

### Study aims

The primary aim of the HEI is to generate a comprehensive biochemical roadmap detailing the human dose-response relationship between exercise and molecular activation. The HEI protocol presented in this report will first be completed with young, sedentary to normally active people, without comorbid diseases. Comprehensive blood profiling will facilitate the identification of circulating blood molecules within major biochemical molecular families (i.e., proteins, polar, moderately polar and lipid metabolites, non-coding RNAs) that are up- or down-regulated by acute bouts of exercise. Secondary aims include: 1.) the determination of the minimal intensity and duration of exercise required to induce a statistically significant change in blood concentration of exercise responsive molecules, 2.) the characterization of dose-response kinetics profiles for responsive molecules, 3.) the identification of immediate *versus* late molecular responses (i.e., molecules up- or down-regulated immediately post exercise *versus* the 60-minute recovery time point) and, 4.) the identification of sex-based differences in the exercise-dose biochemical response.

### Inclusion and exclusion criteria

Phase 1 of the HEI will study healthy, young men and women. Inclusion criteria include (1) age between 20 and 45 years, (2) self-defined white race, (3) absence of established pre-morbid conditions, (4) no active use of tobacco within the last year, and (5) structured weekly exercise that does not exceed more than 150 min of moderate intensity and/or 75 min of vigorous intensity exercise per week as recommended by contemporary physical activity guidelines [2,3,28,29]. Participants with established chronic diseases including hypertension, dyslipidemia, diabetes (type 1 and/or 2), cardiovascular disease, prior or present cancer, lung disease, autoimmune disease, or endocrine dysfunction are not eligible. Additional exclusion criteria encompass pregnancy and the presence of any musculoskeletal condition that would make exercise unsafe or impossible.

### Baseline visit

The experimental protocol will begin with the baseline screening visit during which written informed consent will be obtained prior to any subsequent study activities. Participants will complete the long version of the International Physical Activity Questionnaire (IPAQ), which quantifies physical activity performed over the previous seven days [30], provide detailed information about any routine structured exercise and routine physical activity in other domains of life, and complete a health questionnaire designed to elicit self-reported confirmation of inclusion and exclusion criteria. Subsequently, height and body mass will be measured. A resting 12-lead electrocardiogram (Cardea 20/20™, Cardiac Insight, WA, USA) and a transthoracic echocardiogram (Vivid-q™, GE Healthcare, IL, USA) will be performed to exclude occult forms of cardiac pathology associated with adverse events during exercise. Brachial artery blood pressure will be measured after cardiac assessments to ensure the acquisition of true resting values. Participants will then perform baseline spirometry inclusive of three to five forced vital capacity (FVC) manoeuvres to assess FVC and forced expiratory volume in 1 second (FEV_1_). Following spirometry, participants will perform a maximal effort limited cardiopulmonary exercise test on a motorized treadmill (Woodway Pro XL, Woodway, WI, USA) to determine peak oxygen consumption (V̇O_2peak_) as described in detail below (**see** Cardiopulmonary Exercise Testing). The V̇O_2peak_ protocol and the determination of the target intensities, as described below, will be conducted with each participant walking or running throughout the entire procedure. No walk to run transitions will be permitted. Briefly, the V̇O_2peak_ protocol will begin with a 2-min period at a slow walking (3 km/h) or slow running (~5 km/h) speed depending on participant’s desire to perform subsequent exercise sessions either walking or running. Treadmill speed will then be gradually increased until reaching the velocity that elicits a V̇O_2_ corresponding with 50% of the predicted V̇O_2peak_ derived from the Jones equations [*V˙O*_*2peak*_* = 4.2 - 0.032 x age* (men), *V˙O*_*2peak*_* = 2.6 - 0.014 x age* (women)] [31]. At this point the treadmill speed will be kept constant and the slope will be increased by 0.5% every 15 s, an incremental approach designed to optimize the accuracy of determining sub-maximal ventilatory thresholds, until volitional exhaustion. For participants who elect to walk during the V̇O_2peak_ protocol and in whom a natural walk-run transition is observed prior to reaching 50% of the predicted V̇O_2peak_, the fastest comfortable walking speed will be used for the remainder of the test and the treadmill slope will be subsequently increased as described above. Following ~ 10 min of passive recovery, the V̇O₂/work rate relationship will be determined for use in subsequent experimental visits. This experimental step will ensure that treadmill speed and slope elicit the steady state V̇O_2_ corresponding to values derived from the incremental maximal effort test. To do so, the treadmill speed and grade will be set at the levels corresponding with the V̇O2 at the four different experimental visit target intensities defined below. For each target exercise intensity, treadmill speed and slope will be gradually adjusted until reaching the steady state target V̇O_2_ for 2–4 min.

### Experimental visits

**Fig 1:**
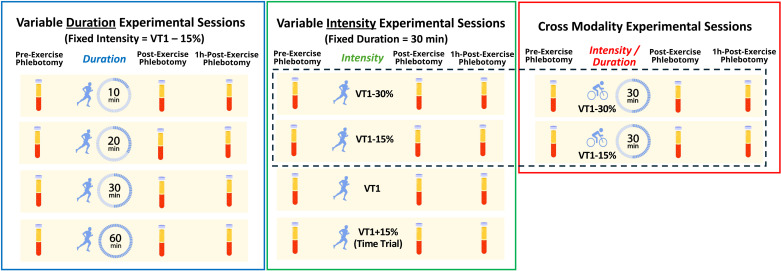
Experimental Visit Exercise Dose Protocol. Participants will perform a total of 10 experimental visits. N = 4 will be dedicated to the manipulation of exercise duration (i.e., variable duration at a constant intensity, *blue* box), N = 4 will be dedicated to the manipulation of exercise intensity (i.e., variable intensity at a constant duration, *green* box), and N = 2 will examine the impact of differential exercise modalities (i.e., up-right cycle ergometer, *red* box).

Participants will perform 10 discrete steady-state exercise sessions, each at a specified exercise dose (Fig 1). Four exercise sessions, performed on the motorized treadmill, will isolate intensity as the modifiable component of exercise dose (i.e., varying intensity at a fixed duration). Four exercise sessions, also performed on the treadmill, will isolate duration as the modifiable component of exercise dose (i.e., varying duration at a fixed intensity). Finally, two sessions will isolate the impact of exercise modality by performing testing on an up-right cycle ergometer (Excalibur Sport, Lode, Netherlands).

The four pre-specified exercise intensities will be defined as the speed and incline, individualized and confirmed for each participant during the baseline visit, required to elicit a percentage of V̇O_2_ at the first ventilatory threshold (VT1) as follows: 1.) V̇O_2_ of 30% below VT1 (VT1–30%), 2.) V̇O_2_ of 15% below VT1 (VT1–15%), 3.) V̇O_2_ of VT1, and 4.) V̇O_2_ of 15% above VT1 (VT1 + 15%). These intensities were chosen to ensure that each participant would be capable of completing at least 30 min of continuous exercise at the three lowest intensities. During the VT1 + 15% visit, participants will exercise to the point of volitional exhaustion. It is anticipated that the majority of participants will reach exhaustion within 30 min under these conditions. In cases where this does not occur, the incline of the treadmill will be gradually increased to ensure that exhaustion is reached within 5 additional minutes.

The four sessions dedicated to the modification of exercise duration will be comprised of fixed intensity exercise at VT1–15% for sessions of 10, 20, 30 and 60 min. This intensity was chosen to ensure that each participant will be capable of completing the longest duration session of 60 min. To examine differential biochemical responses as a function of exercise modality, participants will complete two 30-min sessions on an up-right cycle ergometer at a cadence of 60–80 revolutions per min, each performed at a V̇O2 corresponding to −30% and −15% of treadmill-assessed VT1. Gas exchange will be monitored during the first 5–10 min to ensure that the target V̇O_2_ is reached within 5–6 min. Each session will begin with a continuous 1 W/s ramp until 80% of the target V̇O_2_ is reached, after which individualized manual adjustments will be made to achieve the final workload that correlates with the desired V̇O_2_.

**Fig 2:**
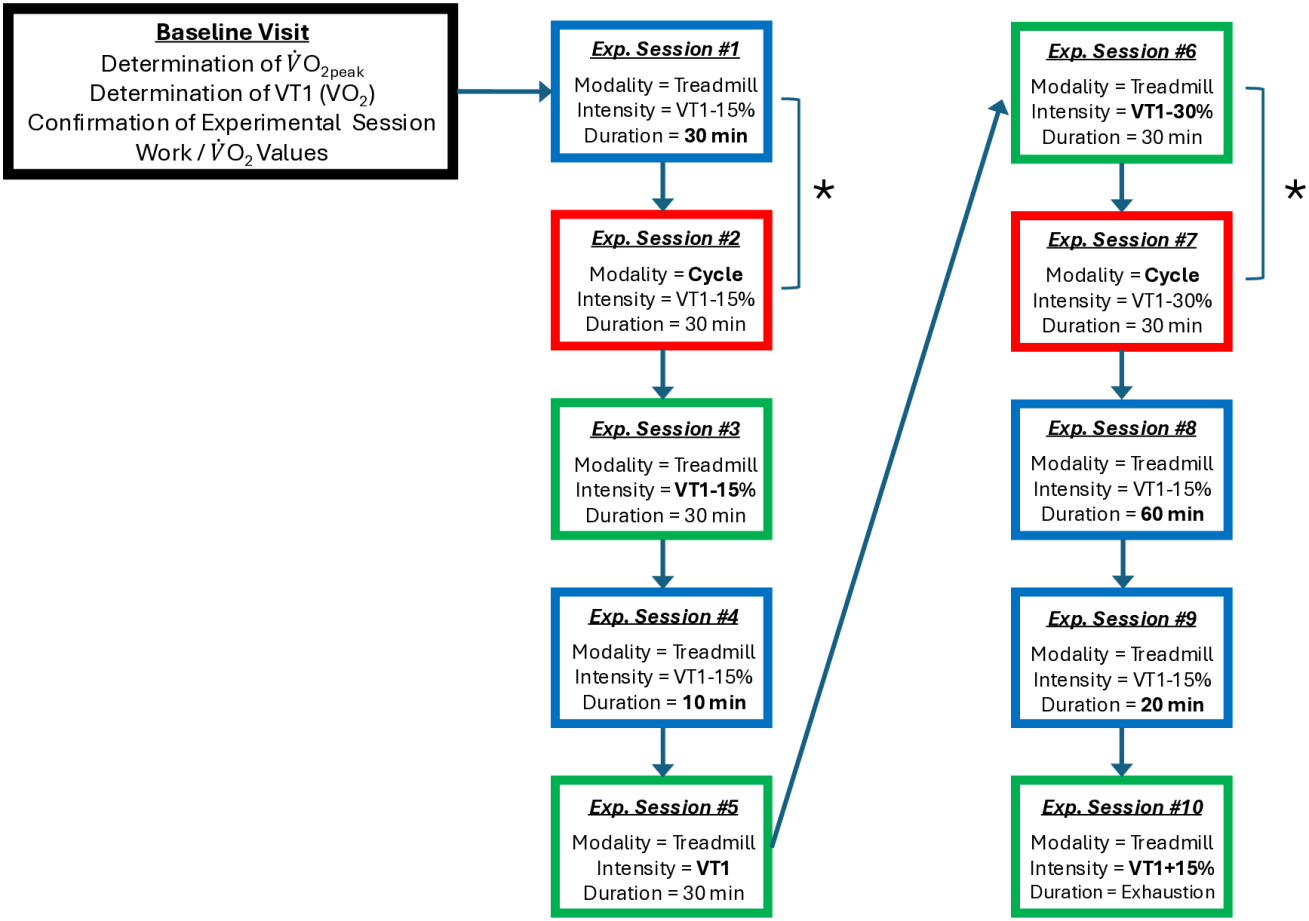
Sequence of Experimental Visits. Following the baseline visit (*black* box), participants will be following the sequence of experimental visits shown here. Experimental sessions designed to manipulate exercise duration (*blue* boxes) will be conducted on the treadmill for: 1.) 10 min, 2.) 20 min, 3.) 30 min, and 4.) 60 min at a fixed exercise intensity of V̇O_2 _= VT1–15%. Experimental sessions designed to manipulate exercise intensity (*green* boxes) will consist of variable intensity treadmill walking/running at V̇O_2 _= 1.) VT1-30%, 2.) VT1-15%, 3.) VT1, and 4.) VT1 + 15% for a fixed duration of 30 min. Modality manipulation (*red* boxes) will include up-right cycling for 30 min at V̇O_2 _= VT1-30% and VT1-15%. * = Direct comparisons to examine differential biochemical responses to running/walking and cycling exercises. VT1 = The first ventilatory threshold.

A predefined sequence of exercise sessions will be applied for all participants (Fig 2). This sequence was implemented to ensure that: 1.) exercise sessions comprised of the higher exercise doses will be performed within the second half of the ten experimental visits, and that 2.) exercise sessions for which direct head-to-head comparisons are planned are not separated by more than one month (e.g., the two identical sessions performed at VT1–15% on the treadmill). To minimize the likelihood and/or impact of a physiologic training effect, consecutive visits will be separated by a minimum of 3 days and participants will abstain from any structured exercise training, above or below their pre-enrollment baseline level of habitual activity, outside of study visits for the duration of study participation.

For each experimental session, participants will report to the laboratory between 7 am and 12 pm following an overnight fast and caffeine intake abstinence. To control for circadian rhythm, all sequential experimental visits for each participant will be scheduled at the same time of the day. Upon arrival, participant body mass and body composition (BC 545, Tanita, Japan) will be recorded. Prior to exercise, an 18–22-gauge medical grade intravenous catheter will be inserted in the most easily accessible superficial peripheral vein at, or distal, to the antecubital fossa. An initial blood sample will be collected immediately prior to exercise initiation. A 5-min warm-up will then be performed at VT1–30% for all experimental sessions performed on the treadmill, or at a gradual intensity starting at 0 W and increasing up to the intensity eliciting the targeted V̇O_2_ for sessions performed on the cycle ergometer. After the 5-min warm-up, the targeted intensity will be maintained for the specific duration of the experimental sessions (i.e., 10–60 min). Immediately at exercise completion, a second blood sample will be collected. Participants will then recover for 60 min prior to the collection of a final blood sample. During the recovery period, all participants will remain seated within the facility and will remain fasted.

### 12-Lead Electrocardiography (ECG)

Resting ECGs will be interpreted by an experienced cardiologist (AB). Quantitative manual measurement of intervals and axes will be performed. Clinical interpretation will include screening for abnormal ECG patterns suggestive of electrical and/or myocardial pathology. Participants with abnormal ECG patterns suggestive of pathology will be referred for clinical evaluation and will not be permitted to continue with study participation until evaluated and deemed appropriate for participation by an unaffiliated cardiovascular specialist.

### Transthoracic Echocardiography (TTE)

TTE image acquisition will be performed in accordance with contemporary clinical guidelines with images acquired from parasternal and apical transducer positions [32]. Quantitative measurements of cardiac structure and function will be performed by an experienced cardiologist (AB). Valve function, screening for stenosis and/or regurgitation, will be assessed in accordance with clinical guidelines [33,34]. Participants with abnormal TTE findings as defined by chamber dilation, chamber hypertrophy, or valvular stenosis and/or regurgitation of greater than mild severity, will be referred for clinical evaluation and will not be permitted to continue with study participation until evaluated and deemed appropriate for participation by an unaffiliated cardiovascular specialist.

### Spirometry

Parameters reflective of pulmonary flows and volumes (FVC, FEV_1_, FEV1/FVC ratio, forced expiratory flow between 25% and 75% of vital capacity (FEF_25–75%_) and peak expiratory flow (PEF)) will be obtained (Quark CPET, COSMED, Italy) in accordance with international standards [35]. Flow–volume tracings from the best of a minimum of three FVC maneuvers will be retained.

### Cardiopulmonary exercise testing

Gas exchange and ventilation parameters will be measured using a commercially available medical grade metabolic cart (Quark CPET, COSMED, Italy). Heart rate will be continuously recorded using a commercial chest strap (Polar H10^TM^, Polar, Finland). The gas analyzer will be warmed up for 30 min and then calibrated according to the manufacturer guidelines. V̇O_2peak_ will be defined as the highest 20-s rolling average value recorded during the incremental cardiopulmonary test [36]. The highest heart rate value recorded will be considered the maximal heart rate (HR_max_). VT1 will be independently determined by two experimenters as the point at which CO_2_ production (V̇CO_2_) increases disproportionately compared to V̇O_2_, marked by a shift in the V̇CO2-V̇O_2_ slope from <1 or 1 to >1 [37]. Confirmation of VT1 will further be confirmed by subsequent evaluation of the relationships between ventilation (V̇E) and workload, V̇E/V̇O_2_ and workload, and P_ET_O_2_ and workload [38,39].

### Blood collection, preparation, and storage

Prior to each exercise session, an 18–22-gauge medical grade catheter (IN400600, BD Venflon^TM^ Pro Safety, BD, NJ, USA) will be inserted in the most easily accessible superficial peripheral vein at, or distal, to the antecubital fossa. During each experimental visit, blood samples will be collected immediately before exercise, immediately after exercise, and after 60 min of passive recovery from exercise. At each time point, approximately 4 mL of blood will be collected for plasma preparation and approximately 4 mL of blood will be drawn for serum preparation. Serum samples will be collected in tubes coated with a clotting activator gel (#01.1602, S-Monovette^®^ Serum Gel CAT, Sarstedt, Germany) and plasma samples in tubes coated with EDTA K3E (# 01.1605.001, S-Monovette^®^, Sarstedt). Tubes will be gently inverted ten times promptly after blood collection. Immediately after collection and prior to centrifugation, plasma tubes will be placed on ice for ∼10 min, while serum tubes will be kept for at least 30 min at room temperature. Both plasma and serum samples will be spun at 2,500 g for 10 min at room temperature. Supernatants will then be aliquoted in 200 μL vials and placed on ice for a maximum of 120 min prior to being stored at −80°C for follow-up analyses. We anticipate that approximately 10 vials of plasma and 10 vials of serum will be placed in frozen storage for each participant time point.

### Data analysis

#### “Omics” analyses.

Multiple *omics* platforms will be used to generate a comprehensive roadmap of the human biochemical exercise dose-response. Specifically, broad-base proteomic, metabolomic, lipidomic and transcriptomic analyses will be performed on plasma samples acquired at each experimental time point. Changes in protein abundance will be determined using liquid chromatography high-field asymmetric-waveform ion-mobility spectrometry tandem mass spectrometry (LC-FAIMS-MS/MS; Thermo Scientific Orbitrap Exploris 480, Thermo Fisher Scientific, California, USA) operated in Data Independent Acquisition mode. Changes in metabolite and lipid levels (i.e., concentrations) will be measured using chromatography – electrospray ionization tandem mass spectrometry approach (LC-ESI MS/MS) in a timed, selected reaction monitoring – t-SRM mode (TSQ Altis Plus, triple-stage quadrupole mass spectrometer, Thermo Fisher Scientific). The metabolite extraction and profiling will be done separately (using separate plasma aliquots) depending on the metabolite/lipid class, and using validated high-coverage targeted methodologies developed within the Metabolomics and Lipidomics Platform of the Faculty of Biology and Medicine of the University of Lausanne [40]. Amino acid, acylcarnitine and steroid quantification will be done using multiple-point calibration with internal standard spike. For complex lipids (~800 species belonging to five major classes including glycerolipids, cholesterol esters, sphingolipids, glycerophospholipids and free fatty acids), the estimated concentrations will be measured using single-point calibration with internal standard spike (75 IS at known concentration using Ultimate Splash mixture, Avanti Lipids) [41]. Transcriptome profiling will be performed using RNAseq technology [42].

#### Statistical analyses.

**Fig 3:**
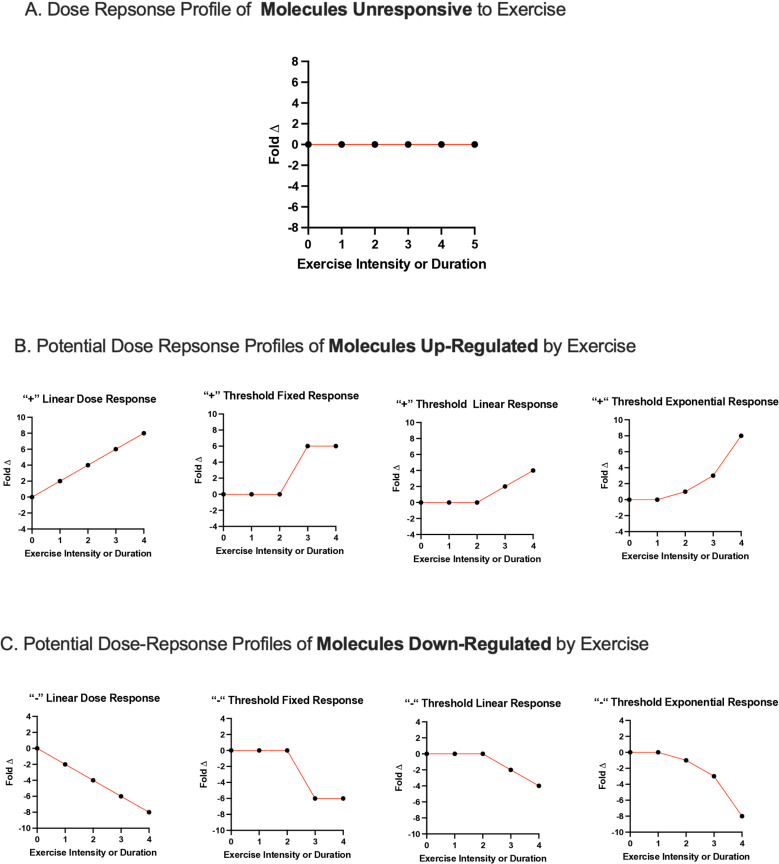
Hypothetical molecular exercise dose-response profiles. It is anticipated that the majority of plasma-based molecules will adhere to one of 3 primary exercise dose-response profiles: *Unresponsive* to exercise (A), *Up-regulated* by exercise (B), or *Down-regulated* by exercise (C).

The Biomedical Data Science Center of the University of Lausanne and the Lausanne University Hospital (Lausanne, Switzerland) will oversee and coordinate all statistical analyses. It is anticipated that this work will generate a large database which will be amenable to conventional biostatistics and to rapidly evolving machine learning techniques. The theoretical basis for the following analyses is shown in Fig 3. Briefly, we hypothesize that measured molecules will conform to one of three specific response profiles: 1) up-regulated molecules, 2) down-regulated molecules, and 3) non-responsive molecules. Among molecules significantly up- or down-regulated by exercise, we further hypothesize that most will exhibit a distinct dose-response pattern, characterized either by a monotonic relationship (e.g., a linear correlation between molecular plasma concentration and exercise intensity and/or duration) or a threshold response. Threshold responsive molecules are anticipated to be unresponsive to low intensity and/or short duration intensity with subsequent up- or down-regulation occurring only in response to higher doses of exercise.

Conventional analyses that will be applied are summarized as follows. The primary goal of the HEI is to identify the exercise intensity and duration dose thresholds of molecular activation or down-regulation. Thus, the primary outcome will be the discrete exercise intensity and duration that produces a statistically different molecular concentration compared to resting baseline conditions. Each participant will undergo 10 discrete resting blood samples (i.e., one prior to each acute bout of exercise). The concentration of each molecule under resting conditions will be used to calculate a participant-specific mean and standard deviation baseline concentration for each molecule. Levels of each molecule will then be similarly measured again from samples obtained immediately and 60-minute after each discrete dose acute exercise session. This will facilitate the generation of immediate post exercise and 60-minute post exercise curves that plot molecular concentration on the Y-axis and exercise dose on the X-axis.

For each participant, the resting molecular concentration will serve as their unique, participant-specific y-intercept, while the rate of change (slope) for each molecule will be assessed during escalating exercise intensity and duration. Mixed-effects linear (or generalized linear) models will be used to identify molecules that significantly change with exercise dose, using slope as a proxy in linear regression, and to determine the precise exercise dose at which the curve deviates from zero. This approach will allow us to pinpoint which molecules respond to exercise and at what intensity or duration each responsive molecule is up-regulated (positive change) or down-regulated (negative change). The potential application of more advanced machine learning techniques (e.g., for dimensionality reduction, regularization, etc.) will be determined in collaboration with the biostatistics team at a later stage, based on the size and characteristics of the final dataset.

#### Sample size justification.

Sample size calculations are based on a proposed sample size of n = 20. Given the time requirements for participants and our prior experience with similar studies, we anticipate a 2:1 attrition rate and have planned for the recruitment of 30 participants. For sample size calculations, alpha was 0.05 and 2-tailed. Power is sensitive to the positive correlation between repeated measures of the outcome variables over time, denoted by ρ, with greater correlation reducing the variance. The plasma protein brain derived neurotrophic factor (BDNF), based on prior work characterizing this protein as being moderately responsive to exercise (i.e., 1.44 ± 0.12 fold change following 30 min of moderate intensity exercise) [24], was used for calculations. We used previously published data to estimate conservatively ρ as 0.6 for the candidate variable [24]. Accordingly, we will have >80% power to detect differences between baseline (resting) and immediate post-peak exercise (post) for the moderately exercise-responsive protein BDNF as low as 1.2-fold change. Importantly this magnitude of change falls well below what we anticipate seeing given the proposed stimulus intensities and duration.

## Discussion

This report summarizes the rationale, study design, and experimental protocols of the Human Exersome Initiative (HEI) which was created with an overarching aim of defining dose-response relationships between exercise and human biochemistry. Coupling carefully controlled laboratory-based exercise sessions at discrete exercise intensities and durations with advanced molecular profiling, we aim to develop a comprehensive list of exercise responsive molecules and to determine the specific intensity and/or duration of exercise required for significant up- or down-regulation. The HEI protocol described here will first be applied to healthy young men and women (Phase 1). Data derived from Phase 1 will broadly define the dose-dependent nature of exercise on human plasma-based biochemistry. We anticipate that data from Phase 1 will facilitate biomarker discovery, molecular pathway discovery, and the identification of optimal exercise doses for use in future studies and clinical interventions.

The broader overarching goal of the HEI is to elucidate how the human exercise dose response varies as a function of human phenotypic variability. Following completion of the initial work focused on young healthy people, we plan to extend this protocol into additional human cohorts including people of older age (65–80 years), people of different ethnicities, trained athletes, and clinical populations with common comorbid diseases. For each of these iterations, Phase 1 data characterizing the molecular exercise response among healthy young people will serve as the comparator for subsequent iterations of the protocol. The identification of how molecular responses differ as a function of human phenotypic variability will provide novel insights into the biochemistry of functional decline (i.e., with older age or the development of common forms of co-morbid disease) and among people with supra-normal exercise capacity (i.e., trained athletes). It is anticipated that these subsequent phases of the HEI will elucidate biochemical mechanisms underlying changes in human exercise capacity across the lifespan and set the stage for the development and refinement of age-specific exercise interventions and guidelines, nutritional supplements, and pharmaceutical compounds that may ultimately attenuate the functional decline.

The HEI was designed to define biochemical responses to acute bouts of exercise. It is recognized that the HEI will not address molecular adaptations to exercise training. Ongoing work within the MoTrPAC will provide important information that will complement data emerging from the HEI [16]. While the rationale and protocol of MoTrPAC have been carefully vetted and described, this project includes a single dose of endurance exercise which may exceed or fall short of exercise doses required for specific biochemical regulation. Accordingly, it is anticipated that the identification of specific exercise doses that are required to stimulate specific molecular responses will permit optimally informed design of future training studies and clinical interventions that will target specific outcomes.
